# IgG-like Bispecific Antibody CD3×EpCAM Generated by Split Intein Against Colorectal Cancer

**DOI:** 10.3389/fphar.2022.803059

**Published:** 2022-02-23

**Authors:** Lei Wang, Yu Qiao, Huifang Zong, Lei Han, Yong Ke, ZhiDi Pan, Jie Chen, Jun Lu, Jinyao Li, Tianlei Ying, Baohong Zhang, Jianwei Zhu

**Affiliations:** ^1^ Engineering Research Center of Cell and Therapeutic Antibody, MOE, School of Pharmacy, Shanghai Jiao Tong University, Shanghai, China; ^2^ Jecho Institute, Co. Ltd., Shanghai, China; ^3^ Jecho Biopharmaceuticals Co. Ltd., Tianjin, China; ^4^ School of Science, and School of Interprofessional Health Studies, Faculty of Health and Environmental Sciences, Auckland University of Technology, Auckland, New Zealand; ^5^ Xinjiang Key Laboratory of Biological Resources and Genetic Engineering, College of Life Science and Technology, Xinjiang University, Urumqi, China; ^6^ Key Laboratory of Medical Molecular Virology (MOE/NHC/CAMS), School of Basic Medical Sciences, Fudan University, Shanghai, China; ^7^ Jecho Laboratories, Inc., Frederick, MD, United States

**Keywords:** colorectal cancer, bispecific antibody, split intein, epcam, CD3

## Abstract

**Background:** Colorectal cancer is a commonly diagnosed cancer with high mortality worldwide. Postoperative recidivation and metastasis still are the main challenges in clinical treatments. Thus, it is urgent to develop new therapies against colorectal cancer. Epithelial Cell Adhesion Molecule (EpCAM) is overexpressed in colorectal cancer cells and strongly associated with cancer development. Bispecific antibody (BsAb) is a kind of promising immunotherapy, which could recognize T cells and cancer cells simultaneously to achieve the anti-tumor effects.

**Methods:** A bispecific antibody targeting EpCAM and CD3 with IgG format was genereated by split intein based on the Bispecific Antibody by Protein Splicing” platform. *In vitro*, the affinity of CD3×EpCAM BsAb was determined by Biolayer interferometry, its cytotoxicity was detected by LDH release assay, T cell recruitment and activation was detected by Flow Cytometry. *In vivo*, its pharmacokinetic parameters were detected, and anti-tumor effects were evaluated on the tumor cell xenograft mouse model.

**Results:** The results showed that the CD3×EpCAM BsAb could activate and recruit T cells via binding colorectal cells and T cells, which could lead to more potent cytotoxicity to various colorectal cell lines than its parent EpCAM monoclonal antibody (mAb) *in vitro*. The CD3×EpCAM BsAb had similar pharmacokinetic parameters with EpCAM mAb and inhibits tumor growth on the SW480 tumor cell xenograft mouse model.

**Conclusion:** The CD3×EpCAM BsAb could be a promising candidate for colorectal cancer treatment.

## Introduction

Colorectal cancer (CRC) is one of the most common cancers with a low 5-years survival rate worldwide (Siegel et al., 2021; Sung et al., 2021). CRC develops from normal mucosa to an invasive tumor through different stages (Argilés et al., 2020). Even though surgery and chemotherapy are standard treatments, chemotherapy could negatively affect normal tissue (Nordlinger et al., 2009). Meanwhile, patients with metastatic colorectal cancer have a poor prognosis, and tumor recurrence is possible within 5 years after surgery (Seo et al., 2013; Van Cutsem et al., 2016). Therefore, a novel therapeutic for colorectal cancer is urgently needed.

In recent years, immunotherapies for CRC have been developed, including bispecific antibodies (BsAb), checkpoint inhibitors, cancer vaccines, and oncolytic virus therapy (Ciardiello et al., 2019). Engineered antibodies are an important part of immunotherapy (Zhu, 2012). The bispecific antibody is an engineering antibody with two different binding targets or epitopes for multiple applications (Brinkmann and Kontermann, 2021). It could exhibit a novel action or mechanism that cannot be achieved by combining separate antibodies with the same targets (Labrijn et al., 2019), such as redirecting T cells to tumor cells (Bargou et al., 2008), activating or inhibiting two receptors (Schaefer et al., 2011), serving as cofactor mimetic (Sampei et al., 2013), or even delivering a protein across the blood-brain barrier (Yu et al., 2011; Yu et al., 2014). Recruiting effector cells to eliminate tumor cells was the typical application of BsAb in immunotherapies. The European Medicines Agency approved the first commercial bispecific antibody targeting CD3 and EpCAM for malignant ascites, followed by FDA approval of Blinatumomab targeting CD3 and CD19 for acute lymphoblastic leukemia, Emicizumab targeting factors IXa and X for Hemophilia A, and Amiivantamab targeting EGFR and cMet for non-small cell lung cancer (Heiss et al., 2010; Blair, 2019; Stein et al., 2019; Neijssen et al., 2021). However, compared to the success of hematological malignancies, solid tumor treatment is still complex in clinical trials.

Important expression characteristics and functional roles contribute to EpCAM (CD326) to an attractive target for immunotherapy (Chaudry et al., 2007; Eyvazi et al., 2018; Song et al., 2021). On the one hand, it is a highly expressed glycoprotein on most carcinomas and slightly expressed on normal tissues. On the other hand, it is crucial for tissue morphogenesis and stable adhesion formation, and it is associated with epithelial cell proliferation, differentiation, and migration (Munz et al., 2004; Went et al., 2006). Compared with the anti-EpCAM monoclonal antibody (de Bono et al., 2004), CD3×EpCAM BsAb (Catumaxomab and MT110) exhibited a better efficacy against EpCAM-positive cancer in clinical trials (Heiss et al., 2010; Münz et al., 2010; Kebenko et al., 2018). Nevertheless, while catumaxomab can drive the immune system to kill tumor cells, it also has serious side effects due to its Fc region’s off-target binding to FcγR^+^ Kuffer cells (Borlak et al., 2016).

The property of the bispecific antibody is deeply affected by the structure and target (Nie et al., 2020). Fragment-based BsAb has intense penetration and epitope accessibility with a short plasma half-life (Fan et al., 2015). For instance, the half-life of Blinatumomab is only 1.25 ± 0.63 h (Klinger et al., 2012). By contrast, IgG-like BsAb with a natural structure could be flexible and desirable for therapeutic clinical application; however, heavy/light chain miss pairing remains an issue. Several alternative strategies were applied to generate bispecific antibodies (Han et al., 2019; Zhou et al., 2020). In our study, the “Bispecific Antibody by Protein Trans-Splicing” (BAPTS) platform was introduced to maintain the correct pairing of chains for the binding affinity of the Fab domain (Han et al., 2017). Split inteins comprise N-terminal fragment (Intein^N^) and C-terminal fragment (Intein^C^) and are inactive when separated. The *trans*-splicing reaction could be carried out under native conditions, including reconstitution of intein fold, excision of the intein, and ligation of exteins. In the BAPTS platform, the bispecific antibody was divided into two antibody fragments attached with Intein^N^ and Intein^C^ at the hinge region of the antibody. The *trans*-splicing reaction occurred between two antibody fragments under mild reducing conditions, resulting in a mature bispecific antibody with correct chain pairing. In addition, the bispecific antibody remains an IgG-like format without an extra linker.

In our previous work, CD3×HER2, CD3×EGFR, HER2×EGFR and CD3×PRLR BsAbs were produced based on the “BAPTS” platform (Han et al., 2017; Zhou et al., 2020). The CD3×EpCAM BsAb was generated with natural human IgG1 structure with the same technology. The CD3×EpCAM BsAb eliminated the tumor cells with high EpCAM expression level, activated T cells and induced T cells redirected to tumor cells *in vitro*. It could inhibit the growth of solid tumors derived from the SW480 cell line and maintain a similar half-life with EpCAM mAb *in vivo*.

## Materials and Methods

### Mice and Cell Culture

Female NOD/SCID mice and Balb/c mice were purchased from Beijing Charles River Laboratories. The experimental protocols were approved by the Institutional Animal Care and Use Committee of Shanghai Jiao Tong University (A2018041). SW480 and Jurkat cells were purchased from the Chinese Type Culture Collection, and Caco-2 and HT-29 cells were purchased from the American Type Culture Collection. All tumor cell lines were cultured according to manufactures’ instructions.

### Transient Antibody Expression in HEK293E Cells

Anti-CD3 and anti-EpCAM variable heavy chain (VH) and variable light chain (VL) sequences were synthesized and extended with human IgG1 framework with mutations of L234A, L235A, and P329G (LALA-PG), and then cloned into expression vector pM09 separately via One Step Cloning Kit (Vazyme Biotech, Nanjing, China) (30). Fragment A contained anti-CD3 light chain, anti-CD3 heavy chain, and inteinC fused CH2-3 construct, and Fragment B contained anti-EpCAM light chain and anti-EpCAM VH and CH1 in tandem with inteinN construct.

Transient gene expression (TGE) was used to produce antibody fragments and monoclonal antibodies as previously described (Ding et al., 2017). In brief, two antibody fragments conjunct with split intein against CD3 and EpCAM were expressed via the transient transfection system in HEK293E cells. Plasmids were prepared through the TM Endo-free Plasmid Maxi Kit (Omega Bio Tek, Norcross, GA, United States). HEK293E cells were seeded at a density of 3×10^6^ cells/mL on the day before transfection and were centrifuged and adjusted at 6×10^6^ cells/mL using Freestyle 293 medium (Thermo Fisher Scientific, Shanghai, China) on the day of transfection. Fragment A and B expression vectors were diluted into 40 μg/ml using freestyle 293 medium and gently mixed with 25 kDa linear-polyethyleneimine (PEI, 1 mg/ml, 23966-1, Polysciences, Warrington, PA, United States) (DNA: PEI = 1:5, *w: w*). Plasmid and PEI complexes were incubated for 10–15 min and added to the cell medium. After 4 h, the transfection system was supplemented with SFM4 HEK293 (Cytiva, Logan, UT, United States) medium equal to freestyle 293 medium in volume and valproic acid (VPA, Sigma Aldrich, Shanghai, China) to 3 mM; after 24 h, 20% Tryptone N1 (Organotechnie, La Courneuve, France) was added into to the culture at 0.5% and anti-clumping agent (Thermo Fisher Scientific, Shanghai, China) to 0.1%. The supernatant was taken for purification when the cell viability was near 50%.

### Bispecific Antibody Generation and Purification

The CD3 and EpCAM fragments were purified by Capto L chromatography (Cytiva, Uppsala, Sweden) through the Akta150 system (Cytiva, Uppsala, Sweden). The supernatant was centrifuged and flirted through a 0.45 μm filter membrane. The column was loaded with binding buffer (20 mM sodium phosphate, 150 mM NaCl, pH 7.4), supernatants, wash buffer (100 mM sodium citrate, pH 5.0), and eluted with elution buffer (100 mM sodium citrate, pH 2.8). The elution was neutralized to pH 7.0 with Tris-HCl buffer (1 M, pH 8.0). Fragment A and B samples were dialyzed into phosphate buffer (PBS, pH 7.4) followed by splicing reaction catalyzed mediated by split intein.

After dialyzing all of the fragments in PBS buffer and adding 2 mM dithiothreitol (DTT, Sigma Aldrich, Shanghai, China), 100 mM fragment A was combined with 375 mM fragment B and incubated at 37°C for 2 h. The mixture was dialyzed to PBS to remove DTT, sterilized using a 0.22 μm filter (Millipore, Shanghai, China), and the oxidation reaction was carried out at room temperature for 2–3 days. The CD3×EpCAM BsAb was isolated through Protein A affinity chromatography (Cytiva, Uppsala, Sweden). The eluates were adjusted to pH 7.0 using Tris-HCl buffer, dialyzed to PBS, and sterilized through a 0.22 μm filter (Millipore, Shanghai, China).

### Affinity Measurement of the CD3×EpCAM BsAb

Biolayer light interferometry (BLI, OctectRED96, ForteBio Analytics, Shanghai, China) was used to evaluate CD3×EpCAM BsAb affinity to EpCAM. Biotinylated EpCAM antigen (Sino Biological, Beijing, China) immobilized to the pre-hydrated streptavidin (SA, 18–5,020, Sartorius) sensors. SA sensors were balanced in PBST +0.1%Tween 20 (assay buffer). Then 50 nM CD3×EpCAM BsAb associated with antigen for 300 s and dissociated in 0.1%PBST for 600 s. The SA sensors were regenerated by 10 mM glycine, pH 3.0 for 10 s, and neutralized in assay buffer for 10 s, repeated three times. CD3×EpCAM BsAb concentration series at 500 nM, 300 nM, 200 nM, 100 nM was measured another four times. The shaking speed was 1,000 rpm. A 1:1 curve fitting model analyzed the association and dissociation results to fit the association constant (k_on_), dissociation constant (k_dis_), and affinity constant (KD).

### *In Vitro* Cytotoxicity Assays

Tumor cells were seeded in culture medium RPMI 1640 (Thermo Fisher Scientific, Shanghai, China) with 10% FBS (Thermo Fisher Scientific, Shanghai, China) at a density of 1×10^4^ cells/well on a 96-well flat-bottom cell culture plate (Corning, New York, NY, United States) and cultured overnight. In RPMI 1640/2% inactivated FBS, 10-fold serial gradient dilution of CD3×EpCAM BsAb was performed starting with a 15 μg/ml concentration. Samples were added to corresponding wells at a final volume of 50 μl. Peripheral blood mononuclear cells (PBMC) were isolated from health volunteers through Ficoll plus (Cytiva, Logan, UT, United States) density centrifugation by standard procedures. In RPMI 1640 with 10% inactivated FBS medium, PBMCs were adjusted to 1×10^5^ cells/well added into the plate at an effector cell: tumor cell (E: T) ratio of 10:1. The cytotoxicity assay was detected after plates were incubated at 37°C for 32–48 h from supernatant samples using CytoTox 96^®^ Non-Radioactive LDH Kit (Promega, Madison, WI, United States). All tests were repeated in triplicate and nonlinear regression analysis to fit dose-response curves and were assayed with GraphPad Prism Version 8.0.

### Analysis of T-Cell Redirection

CD3^+^ Jurkat cells were labeled with PKH26 (Sigma Aldrich, Shanghai, China) as model cells, and SW480 cells were labeled with carboxyfluorescein succinimidyl amino ester (CFSE, Invitrogen, Carlsbad, CA, United States) as target cells, according to manufactures’ instructions. The two kinds of labeled cells were incubated at equal ratios and then added with 150 ng/ml EpCAM mAb, CD3 mAb, CD3×EpCAM BsAb and control antibody for 30 min at 4°C respectively. CFSE^+^/PKH26^+^ cells were detected by flow cytometry.

### Analysis of T-Cell Activation

Freshly prepared peripheral blood mononuclear cells (PBMC) were treated with CD3×EpCAM BsAb, CD3 mAb, EpCAM mAb and control antibody at 150 ng/ml and incubated with or without target cells in 96-well plates. The early activation marker CD69 was detected after 24 h and late activation marker CD25 was detected after 90 h. The PBMC were collected and stained with CD8-FITC (Sino Biological, Beijing, China), CD4-PE (Sino Biological, Beijing, China), and CD69-APC (BD biosciences, San Jose, CA, United States) or CD25-APC (Sino Biological, Beijing, China) by standard flow cytometry.

### Pharmacokinetic Studies

Five male Balb/c mice (6–8 weeks) per group were intraperitoneally (i.p.) treated with CD3×EpCAM BsAb or EpCAM mAb at a dose of 10 mg/kg. Blood samples were diluted 2000-fold for the quantitative assay through enzyme-linked immunosorbent assay (ELISA) standard procedure. In brief, goat anti-human IgG kappa chain-specific antibody (Millipore, Shanghai, China) was coated on 96 well high-affinity protein-binding flat-bottom plates overnight. CD3×EpCAM BsAb or EpCAM mAb were captured by anti-human IgG kappa antibody and then were detected by goat anti-human Fc HRP antibody (Sigma Aldrich, Shanghai, China) for the measurements by TMB solution over OD450 absorption. PK parameters were assayed with a non-compartmental analysis model using the PK solver software.

### Xenograft Tumor for Pharmacodynamics Studies

Human effector cells were isolated from healthy donors using Ficoll density centrifugation. *In vivo* experiments were performed in female NOD/SCID mice (6–8 weeks). Thirty mice were divided into five groups for double-blind and randomized treatment. For each mouse, 6×10^6^ SW480 cells were mixed with 2×10^6^ inactivated PBMCs in a final volume of 100 μl. Each mouse was subcutaneously implanted with PBMC and SW480 cells in an E: T ratio of 1:3 on the right flank. Six mice per group were intraperitoneally injected with CD3×EpCAM BsAb, mAb, and PBS as blank control at indicated doses on the second day. All mice were treated once a week by intraperitoneal administration. Tumor sizes were measured using a caliper every three days and calculated according to the formulation.

$$volume=\left(length\times width^{2}\right)\mathrm{÷2}$$

### Statistical Analysis

The GraphPad was applied to calculate EC_50_ values from nonlinear regression analysis. PK solver calculated the pharmacokinetic parameters using noncompartmental analysis.

## Results

### Designing of Bispecific Antibody Molecule Assembly

**FIGURE 1:**
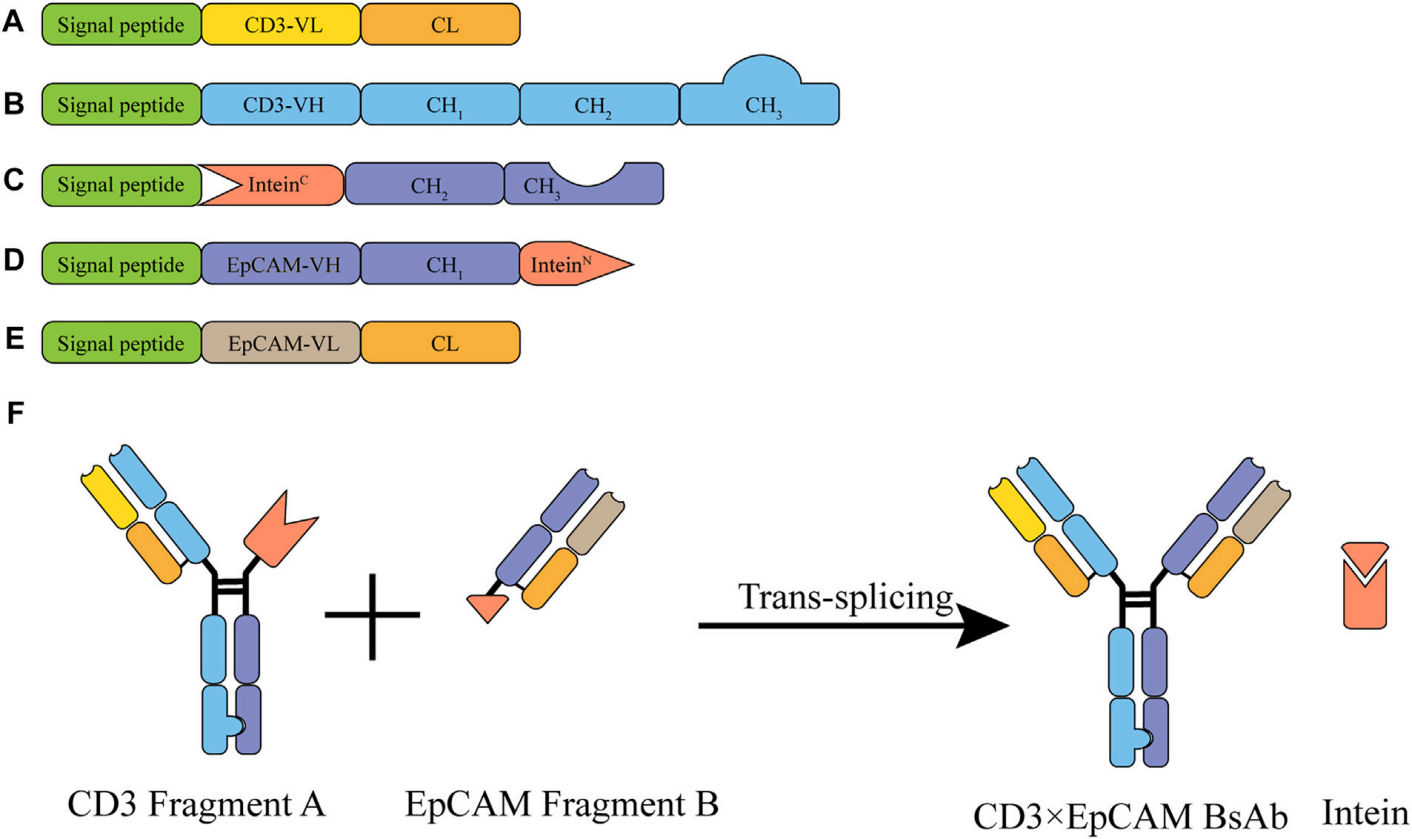
Illustration of the CD3×EpCAM BsAb bispecific antibody generated by BAPTS platform. **(A)** CD3 variable and constant domains for the CD3 light chain. **(B)** CD3 variable and constant domains for the CD3 heavy chain. **(C)** CH2 and CH3 fused with Intein^C^ for Int^c^Fc chain. **(D)** EpCAM variable domain fused with InteinN for HN chain. **(E)** Variable and constant domains for the EpCAM light chain. **(F)** Schematic illustration of CD3×EpCAM BsAb generated by BAPTS platform.

We constructed CD3 and EpCAM fragment expression vectors based on the “BAPTS” platform (Figure 1). Two fragments were expressed in mammalian cells separately (CD3 light chain, CD3 heavy chain, and intein^C^ for fragment A and EpCAM light chain, EpCAM Intein^N^ chain for fragment B. The Antibody-Dependent Cell-mediated Cytotoxicity (ADCC) activity of CD3×EpCAM BsAb was silenced, and “Knobs-into-Holes” were adopted through Fc engineering methods.

### Generation of CD3×EpCAM BsAb

**FIGURE 2:**
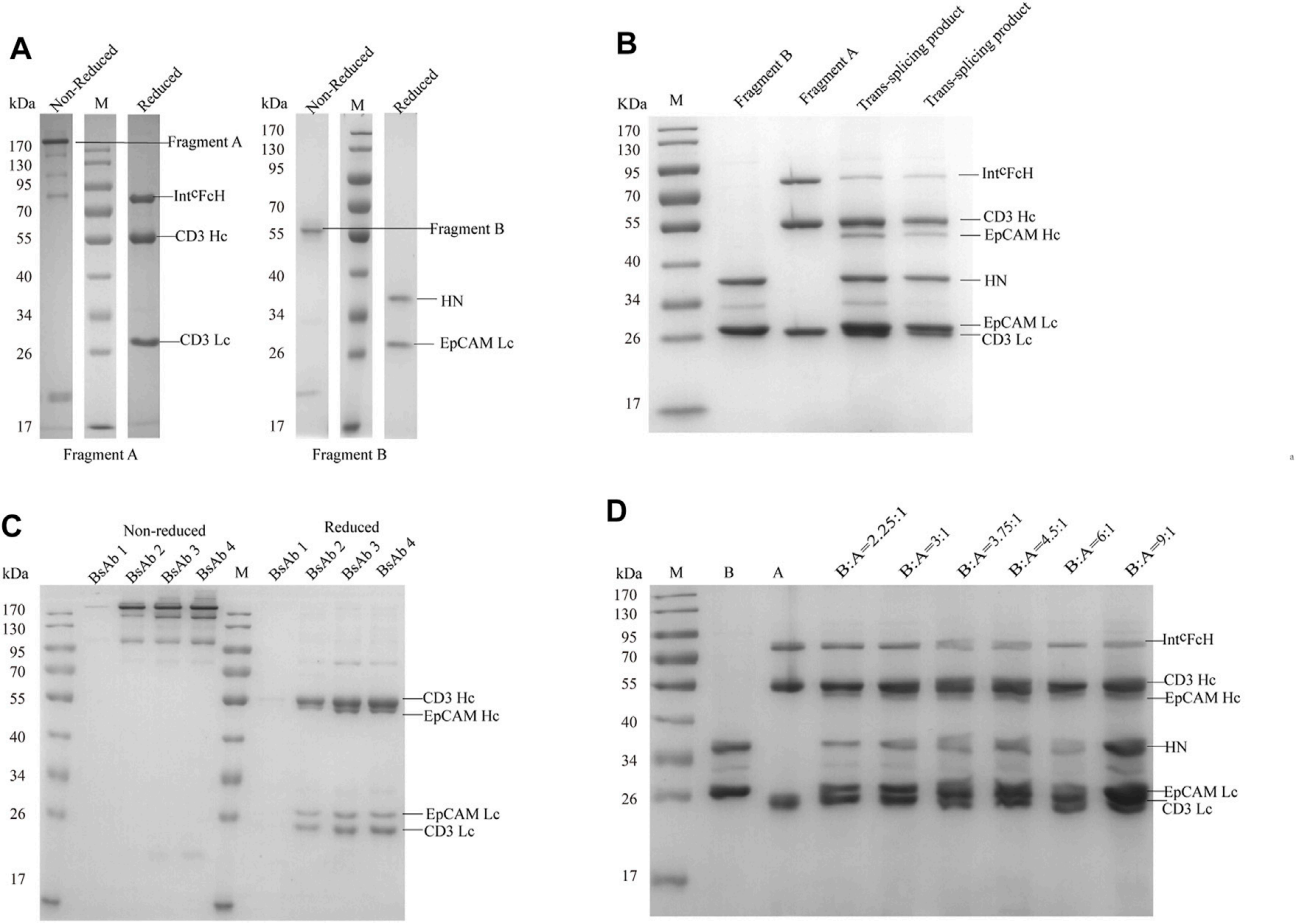
SDS-PAGE analysis of CD3×EpCAM bispecific antibody. **(A)** SDS-PAGE analysis of CD3×EpCAM bispecific antibody CD3 fragment A and EpCAM fragment B under non-reduced and reduced conditions. **(B)** *Trans*-splicing reaction between CD3 fragment A and EpCAM fragment B at 37°C with 1.5 mM DTT for 2 h **(C)** CD3×EpCAM BsAb under non-reduced and reduced conditions. **(D)** *Trans*-splicing reaction condition optimization between the different fragments A and B ratios with 1.5 mM DTT.

After being caught by Capto L affinity chromatography, fragment A and B molecular weight were measured by SDS-PAGE at about 170 and 55 kDa. Fragment A comprised three peptides, CD3 light chain, CD3 heavy chain, and Int^C^FcH chain (Figure 2A, Supplementary Figure S1A). Fragment B comprised two peptides, EpCAM light chain and HN chain (Figure 2A, Supplementary Figure S1B).

When two fragments were reduced with 1.5 mM DTT, the *trans*-splicing reaction mediated by *Npu* DnaE split intein occurred. A new peptide bond was formed in the flanking extein, and SDS-PAGE analysis showed the heavy chain binding to EpCAM at 55 kDa formed (Figure 2B). Then, the dimeric light chain and extra fragment B were removed through Protein A affinity chromatography (Figure 2C). Additionally, a 3.75:1 ratio of fragment B to fragment A (*mol: mol*) was optimized for *trans*-splicing reaction. (Figure 2D). At the same time, an EpCAM parental monoclonal antibody was produced as a control (Supplementary Figure S1C).

### *In Vitro* Cytotoxicity of CD3×EpCAM BsAb

**FIGURE 3:**
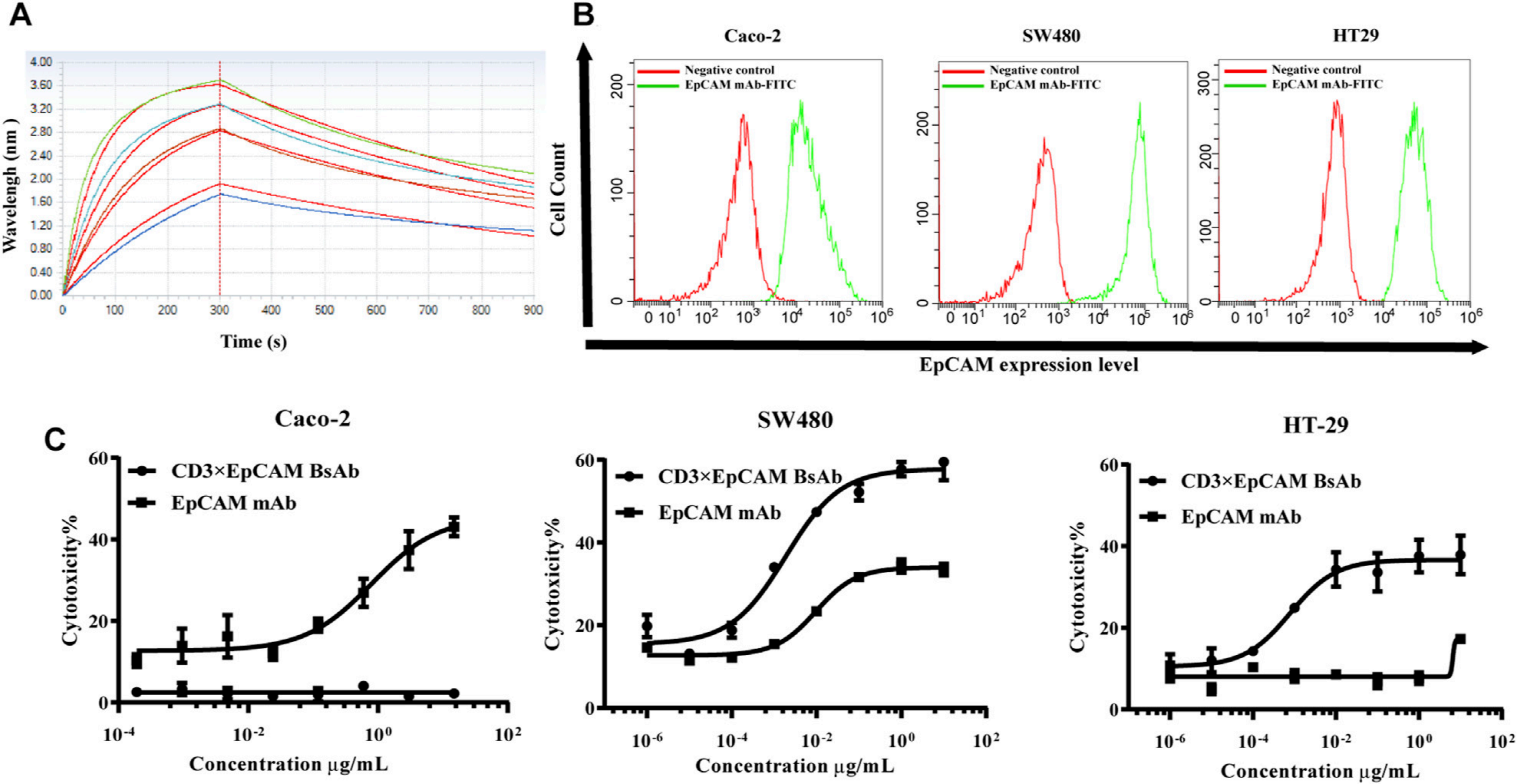
Binding activity to EpCAM and *in vitro* cytotoxicity analysis of CD3×EpCAM BsAb. **(A)** Affinity analysis of CD3×EpCAM BsAb to EpCAM detected by BLI. **(B)** Expression of EpCAM on different cell lines detected by flow cytometry. **(C)** *In vitro* cytotoxicity assay of CD3×EpCAM bispecific antibody using LDH release assay. The dose-response curve was fitted through Graphpad Prism version 8.0. Data points in the curve represent the mean of three samples; error bars, SD.

BLI was used to evaluate the affinity of CD3×EpCAM BsAb for EpCAM. The biotinylated extracellular domain of EpCAM was loaded on the streptavidin sensor and yielded a k_on_ of 2.70 × 10^4^ M^−1^s^−1^, a k_dis_ of 1.05 × 10^−3^ s^−1^, fitting a KD of 3.98 × 10^−8^ M (Figure 3A, Supplementary Figure S2). In addition, the parental antibody was characterized at a KD of nM (Table 1).

**TABLE 1:** Binding kinetics of the CD3×EpCAM BsAb and EpCAM mAb using BLI.

Antibody	KD. (M)	k_on_ (s^−1^)	k_dis_ (M^−1^s^−1^)
EpCAM mAb	3.77 × 10^−12^	1.09 × 10^5^	4.22 × 10^−7^
CD3×EpCAM BsAb	3.98 × 10^−8^	2.70 × 10^4^	1.05 × 10^−3^

Cell surface expression level of EpCAM on our panel of cancer cells was tested using flow cytometry. The highest expression level was Caco-2, followed by HT-29 and SW480. The *in vitro* bioactivity of CD3×EpCAM BsAb was tested using an LDH-release cytotoxicity assay. As effector cells, PBMCs were obtained from healthy donors, and as target cells, a group of tumor cells with different EpCAM expression levels were chosen (Figure 3B). The cells could be killed only in the presence of PMCM and an E:T ratio of 10:1 was taken for the cytotoxicity assay (Supplementary Figure S3A,B). A dose-dependent decrease cytotoxicity effect caused by CD3×EpCAM BsAb was observed at an E:T ratio of 10:1 (Figure 3C). The results showed that tumor cells with the high expression level of EpCAM were sensitive to the BsAb but not to parental EpCAM mAb. And EpCAM negative cell line, Jurkat cell, could not be killed by both CD3×EpCAM BsAb and EpCAM mAb (Supplementary Figure S3C,D). The maximal killing activity of CD3×EpCAM BsAb to different cell lines Caco-2, SW480, and HT-29 were listed (Table 2).

**TABLE 2:** Cytotoxicity analysis of CD3×EpCAM BsAb and EpCAM mAb.

Cell line	Caco-2		HT-29		SW480	
	EpCAM mAb	CD3×EpCAM BsAb	EpCAM mAb	CD3×EpCAM BsAb	EpCAM mAb	CD3×EpCAM BsAb
Top (%)	2.48	45.49	17.29	36.67	33.97	57.79
EC_50_ (ng/ml)	-	797	-	0.78	9.69	1.88

### *In Vitro* T Cell Recruitment and Activation Mediated by CD3×EpCAM BsAb

**FIGURE 4:**
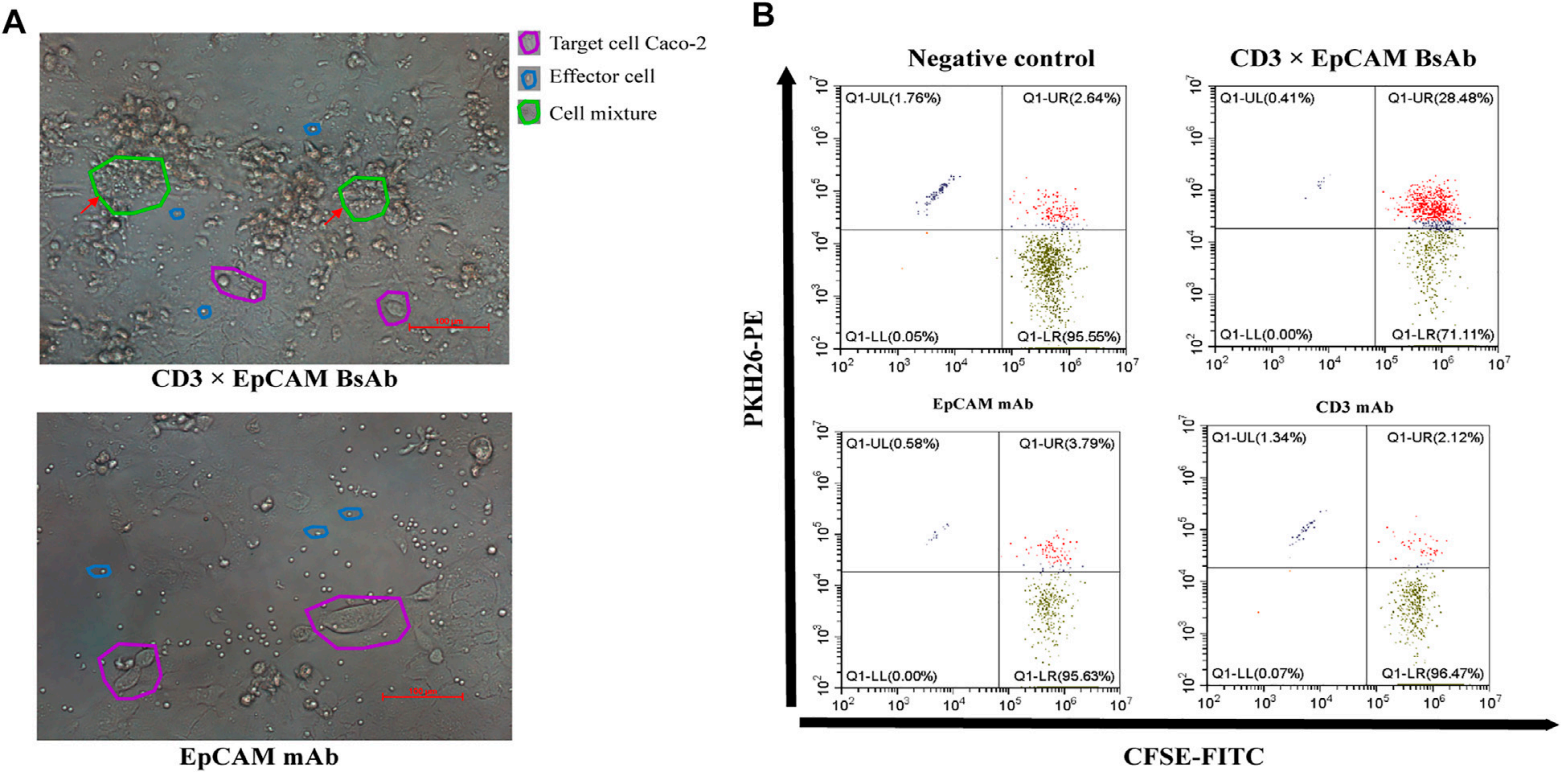
T cell recruitment mediated by CD3×EpCAM BsAb. **(A)** The recruitment of T cells to tumor cells mediated by 150 ng/ml CD3×EpCAM BsAb and EpCAM mAb at an E: T ratio of 10:1 after an incubation of 24 h. Images were obtained under 20 × magnification, and scale bars 100 μm. **(B)** The recruitment analysis of CD3^+^ cells to tumor cells by CD3×EpCAM BsAb by flow cytometry.

The mechanism of CD3×EpCAM BsAb mediated cytotoxicity of cancer cells was investigated (Brischwein et al., 2007; Massafra et al., 2021). The binding ability of CD3×EpCAM to objected cells was tested through flow cytometry. After incubation with CD3×EpCAM BsAb at 100 ng/ml, redirected T cells around tumor cells were observed (Figure 4A), and CFSE^+^/PKH26^+^ cells were more efficiently assembled than CD3 mAb or EpCAM mAb groups. Cell aggression was observed, indicating that compared with EpCAM mAb or CD3 mAb, T cells were significantly redirected to tumor cells by CD3×EpCAM BsAb. (Figure 4B).

**FIGURE 5:**
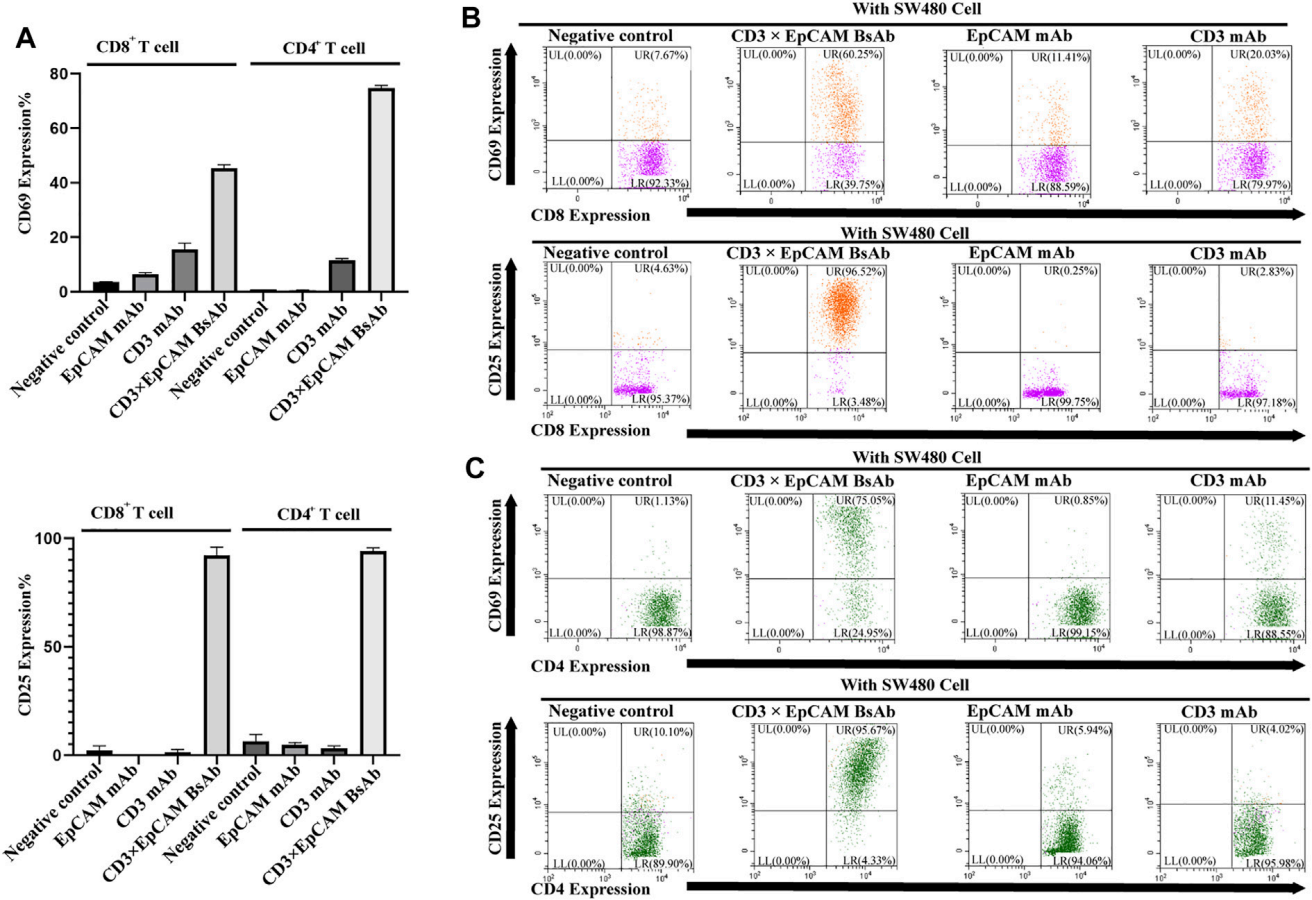
T cell activation analysis mediated by CD3×EpCAM BsAb detected by flow cytometry. **(A)** The activation expression level of CD69 on T cells was detected after incubation for 24 h and CD25 for 90 h (E: T = 10:1). **(B)** The activation analysis of CD8^+^ cells to tumor cells by CD3×EpCAM BsAb, EpCAM mAb, and CD3 mAb. **(C)** The cell activation analysis of CD4^+^ cells to tumor cells by CD3×EpCAM BsAb, EpCAM mAb, and CD3 mAb.

Meantime, the early activation marker CD69 and late activation marker CD25 of CD8 or CD4 positive T cells was also determined by flow cytometry. In the presence of the target cell, activation maker CD69 of T cells was upregulated obviously by CD3×EpCAM BsAb (Figure 5A). In comparison to CD3 mAb or EpCAM mAb, CD3×EpCAM BsAb efficiently activated T cells, resulting in increased CD69 and CD25 expression on CD8^+^ and CD4^+^ T cells (Figures 5B,C). The results also showed the CD107a could be upregulated by CD3×EpCAM BsAb on CD4^+^ and CD8^+^ T cells (Supplementary Figure S4).

### *In Vivo* Bioactivity Study

**FIGURE 6:**
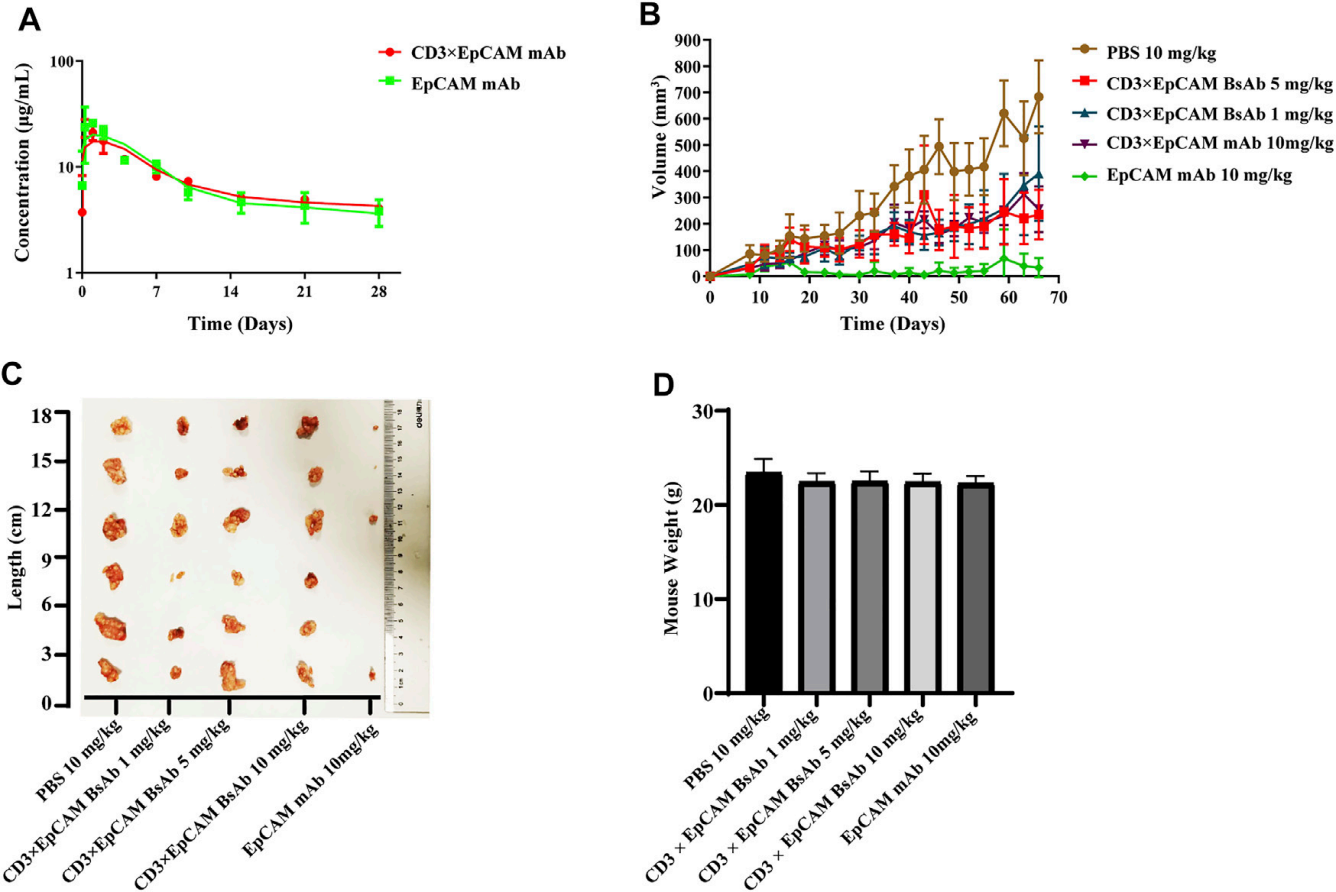
*In vivo* bioactivity assay of CD3×EpCAM BsAb in xenograft NOD/SCID mouse model. **(A)** *In vivo* PK analysis of CD3×EpCAM BsAb and EpCAM mAb in Balb/c mice (N = 5). **(B)** Tumor growth inhibition efficacy of CD3×EpCAM BsAb. Mice were implanted subcutaneously with 6×10^6^ SW480 cells mixed with 2×10^6^ unstimulated human PBMC (E: T = 1:3). Mice (N = 6) were treated through intraperitoneal injection with PBS vehicle control, EpCAM mAb, or CD3×EpCAM BsAb at different dosages per mouse per week. **(C)** Excised tumor results showed on double-blind order. **(D)** Weight comparison of different groups. Error bar, SD.

We performed PK and PD experiments to investigate the *in vivo* bioactivity of CD3×EpCAM BsAb. Balb/c mice were i. p. treated with a single dose of 10 mg/kg CD3×EpCAM bispecific antibody or parental EpCAM mAb, respectively. The blood samples were gathered at a series of time points and quantified by Elisa assay (Figure 6A). CD3×EpCAM bispecific antibody parameters were consistent with EpCAM mAb as excepted (Table 3). It is worth noting that the CD3×EpCAM bispecific antibody was stable with an *in vivo* half-life of about 14 days. The CD3×EpCAM BsAb is lack of cross-reactivity to muEpCAM, so the EpCAM related toxicity and elimination could not be reflected in the mouse model.

**TABLE 3:** PK parameters of CD3×EpCAM BsAb and EpCAM mAb.

	CD3×EpCAM BsAb	EpCAM mAb
CL (mL/day/kg)	0.032	0.026
AUC (day × μg/mL)	229.42	275.56
Cmax (μg/ml)	28.68	27.31
Vz (ml/kg)	0.69	0.57
t _1/2_ (day)	14.89	14.79

The anti-tumor bioactivity of CD3×EpCAM BsAb was evaluated on xenograft NOD/SCID mouse model. The NOD/SCID mice were implanted subcutaneously with a 3:1 mixture of SW480 cells and unstimulated human PBMCs and treated with CD3×EpCAM BsAb or EpCAM mAb through intraperitoneal injection every week. After 66 days, the results revealed that the experimental groups could reduce the growth of the SW480 tumor, although there was no dose-dependent inhibition in the 1 mg/kg, 5 mg/kg, and 10 mg/kg groups (Figures 6B,C). Unexpectedly, EpCAM mAb at a dosage of 10 mg/kg resulted in considerable tumor elimination. In addition, as a sign of toxicity, there was a weight loss of mice in both CD3×EpCAM BsAb and EpCAM mAb groups (Figure 6D).

## Discussion

Colorectal cancer is still a commonly diagnosed cancer with high mortality (Arnold et al., 2017), and an increasing number of novel therapeutics were explored into the clinical stage. This study generated a kind of IgG format bispecific antibody targeting CD3 and EpCAM without an extra linker based on the “BAPTS” platform. Furthermore, the CD3×EpCAM BsAb characterizations *in vitro* and *in vivo* bioactivity, specificity, affinity, and stability were demonstrated.

EpCAM is an attractive target overexpressed on colorectal tumor cells and slightly expressed on normal tissues (Moldenhauer et al., 1987). In order to evaluate the safety, tolerability, pharmacokinetics and pharmacodynamics of solitomab (MT110, AMG 110), a phase I trial in patients with advanced solid tumors was investigated. Diarrhea and transient abnormal liver parameters were dose-limiting toxicities (DLT), leading to a maximum tolerated dose of 48 μg/d (Kebenko et al., 2018). It’s noticed that EpCAM-related toxicity on non-pathological epithelial tissue, especially like hepatocytes and duodenal, when design an anti-EpCAM-antibody based therapy (Schmelzer and Reid, 2008). Besides, a half-life-extended bispecific T-cell engager molecule format was developed by Amgen, which possessed of the Fc region and may change the administration method (Giffin et al., 2021). CD3×EpCAM BsAb exhibited superior cytotoxicity than EpCAM mAb on colorectal cell lines with different EpCAM expression levels *in vitro* (Figure 3B). For safety reasons, the affinity to EpCAM of CD3×EpCAM BsAb was the nM scale in our study (Figure 3A), which could elicit a robust and toxic immune response when binding to EpCAM positive cells (Ruf et al., 2007). The redirection and activation of T cells were confirmed. T cells were bridged with tumor cells through the BsAb rather than the parent monoclonal antibody. T cells were activated by binding of CD3ε without major histocompatibility complex (MHC) restriction, leading to the increased release of granzyme B and perforin (Offner et al., 2006; Bargou et al., 2008). More research could investigate co-simulation or co-inhibitory receptor expression after bispecific antibody treatment to better understand the T activation and cytotoxicity mechanism (Skokos et al., 2020). SW480 xenograft mouse model was established to evaluate the efficacy of CD3×EpCAM BsAb (Schlereth et al., 2005). The growth of the tumor in the NOD/SCID model was inhibited under different dosages of CD3×EpCAM BsAb (Figure 6B). The anti-tumor response among different dosages of CD×EpCAM BsAb was similar in the early stage, while 10 mg/kg CD3×EpCAM BsAb group did not show a superior *in vivo* bioactivity. We considered it might be because the extra CD3 fragment blocked the CD3 antigen on human PBMC in the system or T cell exhaustion on the early stage of administration (Wherry and Kurachi, 2015). The NOD/SCID mouse was widely used in the pre-clinical stage of immunotherapy (Amann et al., 2009; Chen et al., 2021; Sun et al., 2021). However, human immune cells with a limited half-life in the treatment cycle might hard to infiltrate into tumor tissues (Shultz et al., 2005). As the bispecific antibody is specific for human EpCAM and could not bind muEpCAM expressed on mouse normal tissues, the toxicity and distribution could not be reflected in the mouse model. It was worth mentioning that the 10 mg/kg EpCAM mAb group eliminated the tumor growth on the xenograft mouse model. That might be due to EpCAM mAb with low dissociation constant to EpCAM (kdis = 10^−7^ s^−1^) could specifically target tumor cells and then lead to a more prolonged and robust ADCC activity in this model. EpCAM mAb processing bivalent antigen-binding domain could bind to EpCAM more easily than CD3×EpCAM BsAb with single binding valency in this model. Thus, bispecific antibodies with bivalent binding domain to tumor antigen might be more potent to eliminate tumors (Santich et al., 2020). Immune checkpoint inhibitors, including cytotoxic T cell lymphocyte antigen-4 antibody and programmed death-1 antibody, could block the receptor to down-regulate T cells activation and were used to treat colorectal cancer (Chang et al., 2017; Pan et al., 2021). Combination CD3×EpCAM BsAb with the immune checkpoint inhibitor with CD3×EpCAM BsAb might be more helpful to improve the anti-tumor efficacy in future work.

## Data Availability

The original contributions presented in the study are included in the article/Supplementary Material, further inquiries can be directed to the corresponding author.

## Supplementary Material

Supplementary Figure S1
**(A)** CD3 fragment A the EpCAM fragment B under non-reduced and reduced conditions. **(B)** EpCAM fragment B under non-reduced and reduced conditions. **(C)** EpCAM mAb under non-reduced and reduced conditions. Lanes: 1, cell culture supernatant; 2, protein L flow through; 3 to 6, different fractions of protein L elution. Lanes 7 to 12 are the corresponding samples of lanes 1 to 6 analyzed under reduced condition. Lanes 13 to 15, different fractions of protein A elution. Lanes 16 to 17 are the corresponding samples of lanes 13 to 15 analyzed under reduced condition.

Supplementary Figure S2 Subtracted data process of CD3×EpCAM BsAb in the binding test to EpCAM.

Supplementary Figure S3
*In vitro* cytotoxicity assay of EpCAM negative cells and optimization. **(A)** *In vitro* cytotoxicity assay in the presence of T cells and target cells. **(B)** *In vitro* cytotoxicity assay of CD3×EpCAM BsAb to SW480 cells at a concentration of 1.5 mg/ml in different E:T ratio. **(C)** Expression of EpCAM on Jurkat cells detected by flow cytometry. **(D)** *In vitro* cytotoxicity assay of CD3×EpCAM BsAb and EpCAM mAb to Jurkat cells detected by LDH release assay.

Supplementary Figure S4 T cell activation maker CD107a analysis detected by flow cytometry. **(A)** The CD107a detection of CD8^+^ cells to tumor cells by CD3×EpCAM BsAb, EpCAM mAb, and CD3 mAb. **(B)** The CD107a detection of CD4^+^ cells to tumor cells by CD3×EpCAM BsAb, EpCAM mAb, and CD3 mAb.
