# Supplementary figures and images for: IgG-like Bispecific Antibody CD3×EpCAM Generated by Split Intein Against Colorectal Cancer
Source: Front Pharmacol. 2022 Feb 23;13:803059. doi: 10.3389/fphar.2022.803059 (PMC8905292; doi:10.3389/fphar.2022.803059)

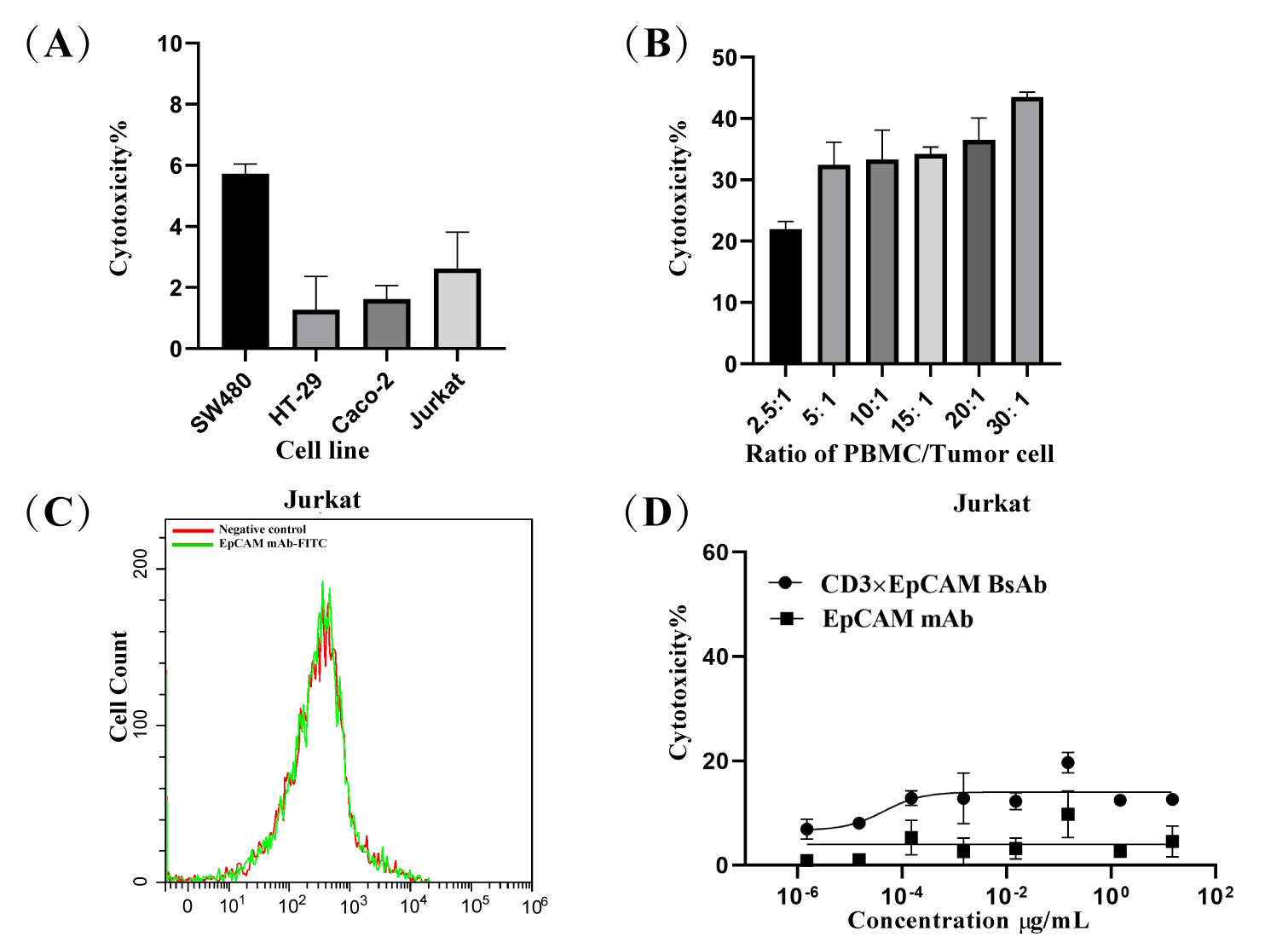

Supplement: Supplementary file 1 [file Image3.tif]

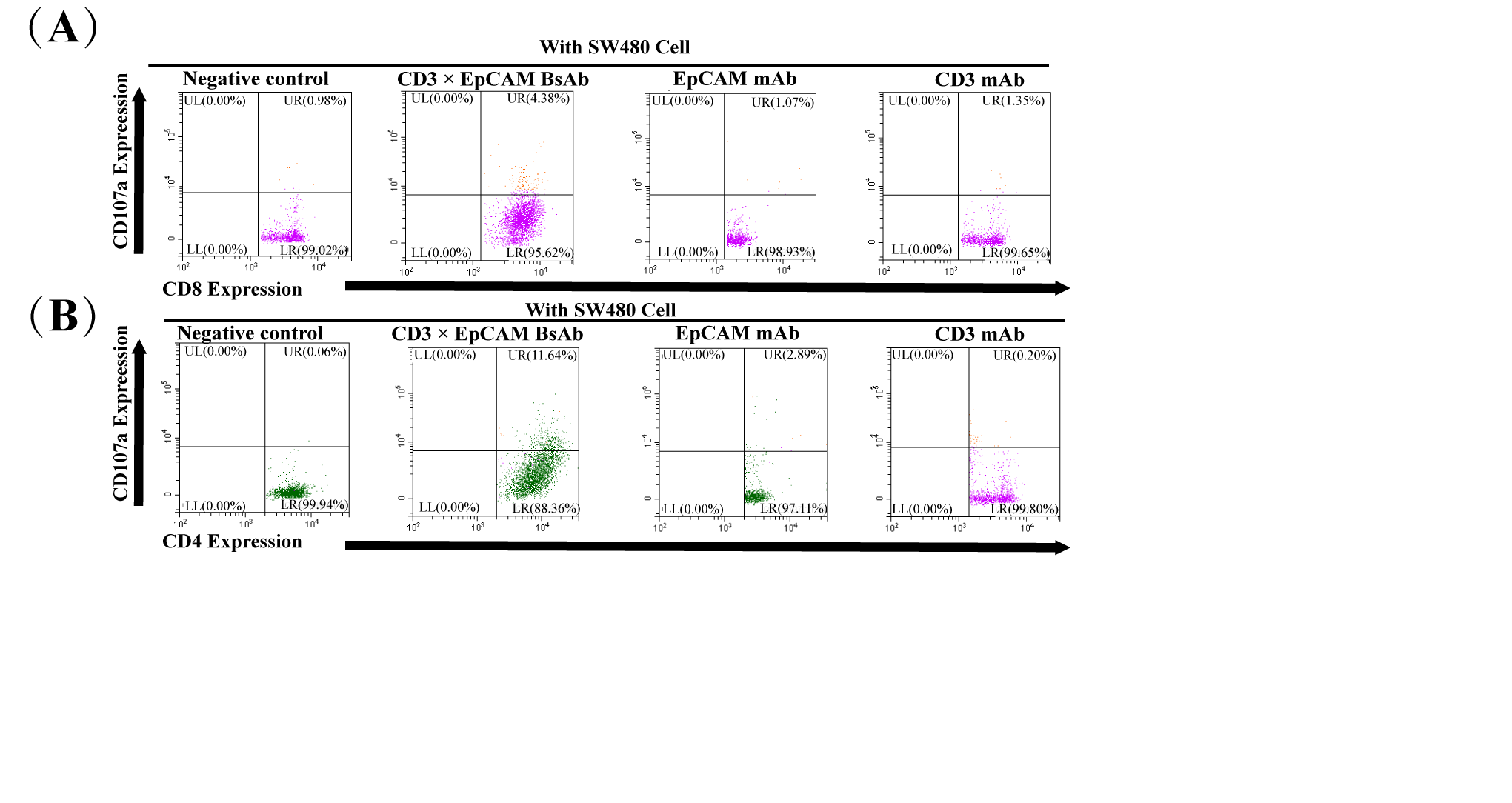

Supplement: Supplementary file 2 [file Image4.tif]

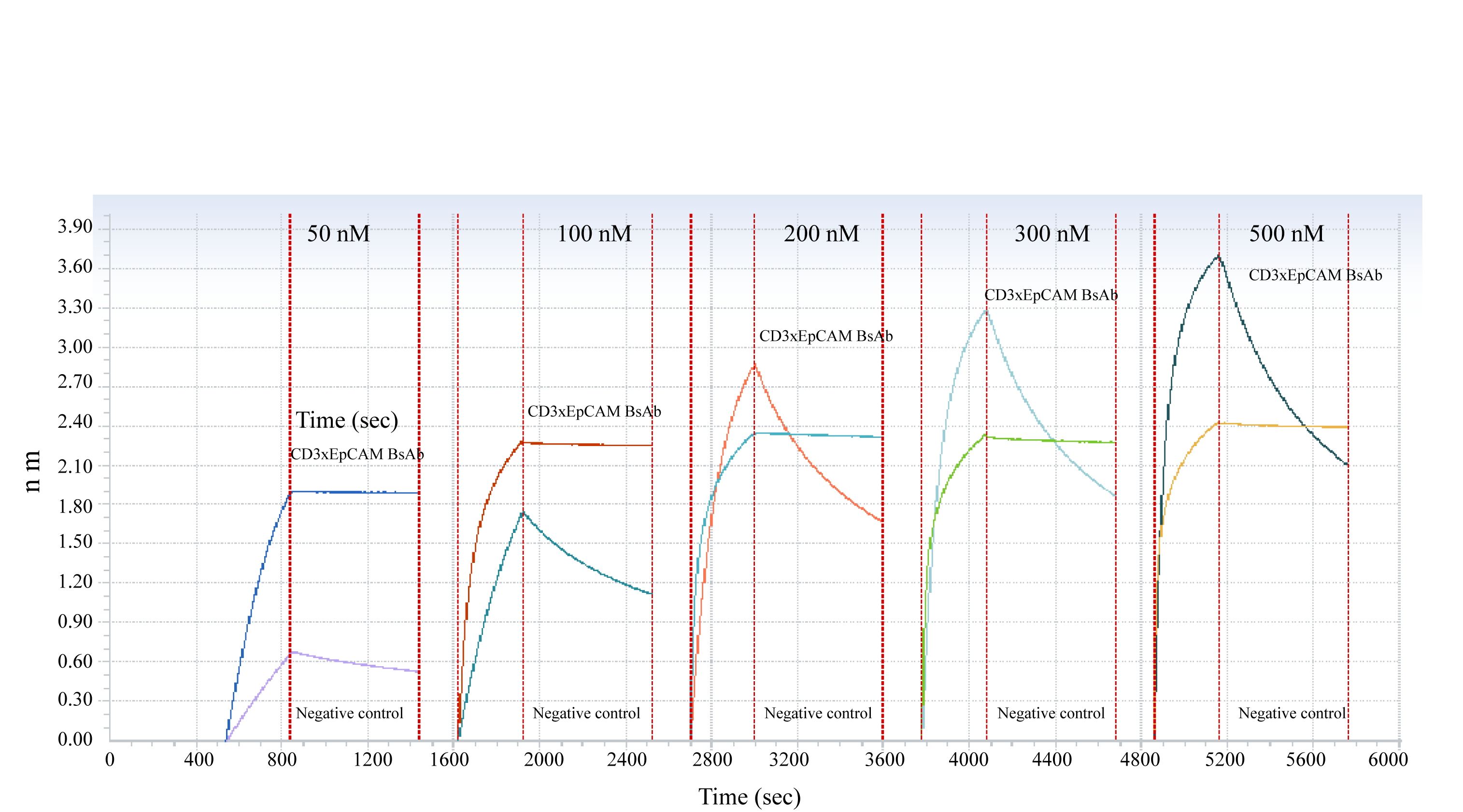

Supplement: Supplementary file 3 [file Image2.tif]
