# Supplementary material for: IgG-like Bispecific Antibody CD3×EpCAM Generated by Split Intein Against Colorectal Cancer
Source: Front Pharmacol. 2022 Feb 23;13:803059. doi: 10.3389/fphar.2022.803059 (PMC8905292; doi:10.3389/fphar.2022.803059)
Supplement: Supplementary file 4 [file Image1.pdf]

Supplementary Material

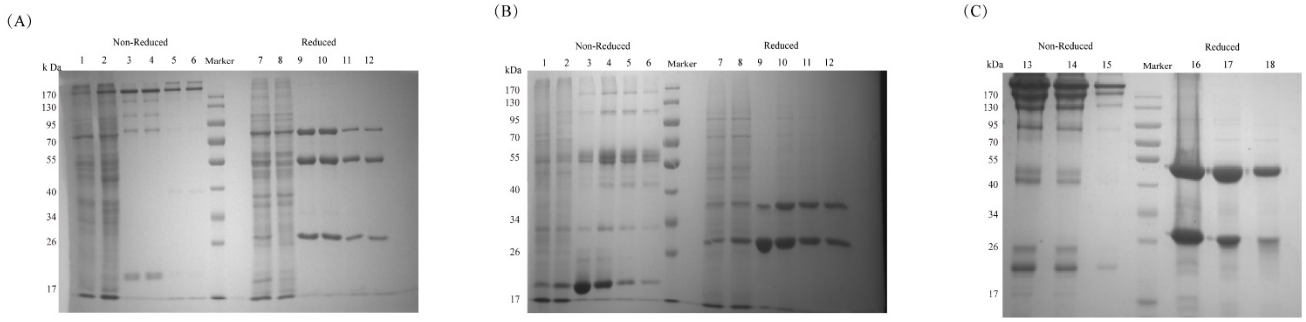

Supplementary Figure 1. (A) CD3 fragment A the EpCAM fragment B under non-reduced and reduced conditions. (B) EpCAM fragment B under non-reduced and reduced conditions. (C) EpCAM mAb under non-reduced and reduced conditions. Lanes: 1, cell culture supernatant; 2, protein L flow through; 3 to 6, different fractions of protein L elution. Lanes 7 to 12 are the corresponding samples of lanes 1 to 6 analyzed under reduced condition. Lanes 13 to 15, different fractions of protein A elution. Lanes 16 to 17 are the corresponding samples of lanes 13 to 15 analyzed under reduced condition.
